# Supplementary material for: Functional Characterization of Novel Bony Fish Lipoxygenase Isoforms and Their Possible Involvement in Inflammation
Source: Int J Mol Sci. 2022 Dec 16;23(24):16026. doi: 10.3390/ijms232416026 (PMC9787790; doi:10.3390/ijms232416026)
Supplement: Supplementary file 1 [file ijms-23-16026-s001.zip › ijms-2042103-supplementary Figure S1.pdf]

International Journal of *Molecular Sciences*

MDPI

Supplemental Material to the paper

Functional characterization of novel bony fish lipoxygenase isoforms and their possible involvement in inflammation

Sophie Roigas, Dagmar Heydeck and Hartmut Kuhn *

Department of Biochemistry, Charité - Universitätsmedizin Berlin, Corporate member of Freie Universität Berlin and Humboldt Universität zu Berlin, Chariteplatz 1, D-10117 Berlin, Germany

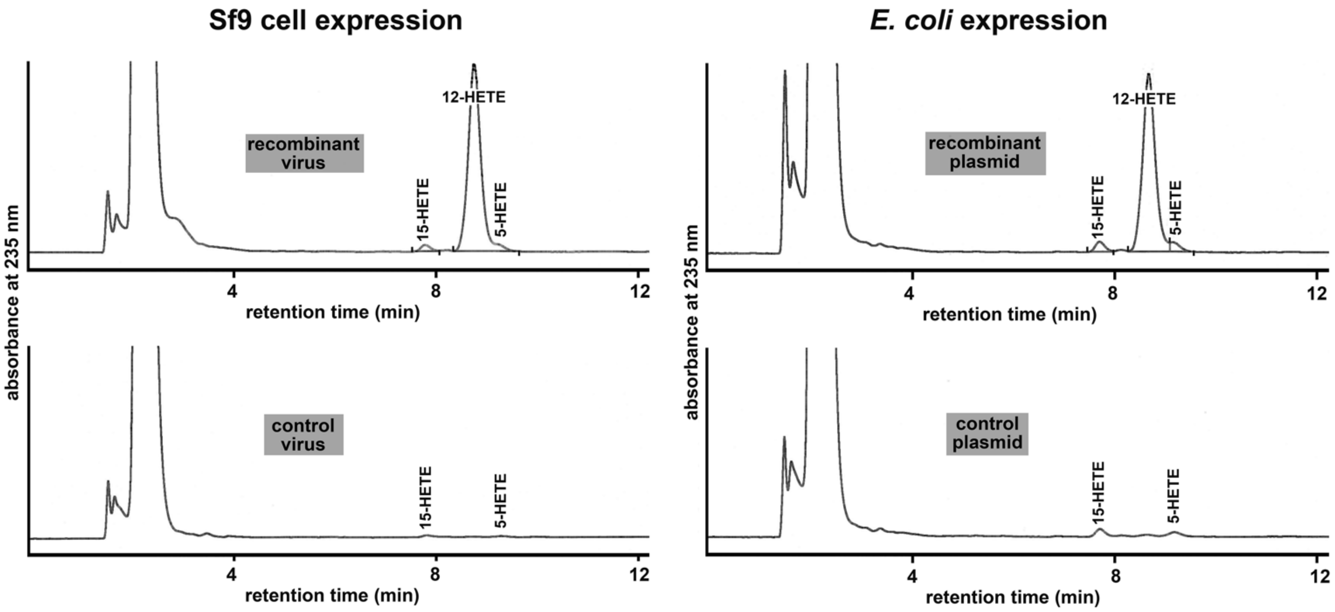

Figure S1. Expression of the putative ALOX15 orthologs of *N. furzeri* (Sf9 cells) and *S. formosus* (*E. coli*). The putative ALOX15 orthologs were expressed as recombinant N-terminal his-tag fusion proteins in Sf9 insect cells or *E. coli* as described in the Materials and methods section(upper panels). For control experiments (lower panels) Sf9 cells were infected with a non-lipoxygenase containing baculovirus and cellular lysate supernatants of these cells were employed for the activity assays. Similar control incubations were carried out with a cellular lysate supernatant prepared from E. coli cells transformed with a no-lipoxygenase containing expression plasmid
