# Supplementary material for: Functional Characterization of Novel Bony Fish Lipoxygenase Isoforms and Their Possible Involvement in Inflammation
Source: Int J Mol Sci. 2022 Dec 16;23(24):16026. doi: 10.3390/ijms232416026 (PMC9787790; doi:10.3390/ijms232416026)
Supplement: Supplementary file 1 [file ijms-23-16026-s001.zip › ijms-2042103-supplementary Table S1.pdf]

Tabel S1: Number of species evaluated in relation to the overall number of described species, and numbers of threatened species by major groups of organisms.

	Estimated Number of described species[1]	Number of species evaluated by 2022 (IUCN Red List version 2022-1)	% of described species evaluated by 2022 (IUCN Red List version 2022-1)	Number of threatened species[2] by 2022 (IUCN Red List version 2022-1)	Estimated % threatened species in 2022 (IUCN Red List version 2022-1)[2,3,4]		
					Lower estimate (threatened spp. as % of extant evaluated species)	Best estimate (threatened spp. as % of extant data sufficient evaluated species)	Upper estimate (threatened and DD spp. as % of extant evaluated species)
**VERTEBRATES**							
Mammals [5]	6,577	5,969	91%	1,337	23%	26%	37%
Birds	11,162	11,162	100%	1,409	13%	13%	13%
Reptiles	11,690	10,150	87%	1,845	18%	21%	33%
Amphibians	8,463	7,316	86%	2,515	35%	41%	51%
Fishes	36,248	24,356	67%	3,548		*Insufficient coverage*	
**Subtotal**	**74,140**	**58,953**	**80%**	**10,654**			
**INVERTEBRATES**							
Insects	1,053,578	12,161	1.2%	2,291		*Insufficient coverage*	
Molluscs	84,528	9,017	11%	2,384		*Insufficient coverage*	
Crustaceans[6]	80,122	3,197	4%	745		*Insufficient coverage*	
Corals	5,574	846	15%	232		*Insufficient coverage*	
Arachnids	110,615	441	0.40%	251		*Insufficient coverage*	
Velvet Worms	210	11	5%	9		*Insufficient coverage*	
Horseshoe Crabs	4	4	100%	2	50%	100%	100%
Others	157,543	904	0.57%	152		*Insufficient coverage*	
**Subtotal**	**1,492,174**	**26,581**	**2%**	**6,066**			
**PLANTS** [7]							
Mosses [8]	21,925	282	1.3%	165		*Insufficient coverage*	
Ferns and Allies [9]	11,800	747	6%	288		*Insufficient coverage*	
Gymnosperms	1,113	1,046	94%	436	42%	42%	44%
Flowering Plants	369,000	59,222	16%	23,551		*Insufficient coverage*	
Green Algae [10]	12,382	16	0.1%	0		*Insufficient coverage*	
Red Algae [10]	7,480	58	0.8%	9		*Insufficient coverage*	
**Subtotal**	**423,700**	**61,371**	**14%**	**24,449**			
**FUNGI & PROTISTS** [11]							
Lichens	17,000	86	0.5%	62		*Insufficient coverage*	
Mushrooms, etc.	120,000	511	0.4%	222		*Insufficient coverage*	
Brown Algae [10]	4,485	15	0.3%	6		*Insufficient coverage*	
**Subtotal**	**141,485**	**612**	**0.4%**	**290**			
**TOTAL**	**2,131,499**	**147,517**	**7%**	**41,459**			

NOTES:

1. The numbers of described species in Table 1a should be used with caution as these are not always be up to date for all taxonomic groups. The sources used for the figures currently shown in the table are listed below.

2. Threatened species are those listed as Critically Endangered (CR), Endangered (EN) or Vulnerable (VU).

3. Where <80% of species within a group have been evaluated, figures for % threatened species are not provided because there is insufficient coverage for these groups. It is only possible to provide reliable figures for % threatened species for those groups that are completely or almost completely evaluated (e.g., mammals, birds, amphibians and gymnosperms).

4. The percentage of threatened species can be calculated for those groups that are completely or almost completely evaluated (>80% of species evaluated), but the actual number of threatened species is often uncertain because it is not known whether Data Deficient (DD) species are actually threatened or not. Therefore, a range of percentages is provided: **lower estimate** = % threatened extant species (if all DD species are not threatened); **best estimate** = % threatened extant species (if DD species are equally threatened as data sufficient species); **upper estimate** = % threatened extant species (if all DD species are threatened). If a single figure is required for reporting purposes, the best estimate figure should be used.

5. The number of described and evaluated mammals excludes domesticated species like sheep (*Ovis aries*), goats (*Capra hircus*), Dromedary (*Camelus dromedarius*), etc.

6. Crustaceans include six classes: Branchiopoda (fairy shrimp, clam shrimp, etc.); Cephalocardia (horseshoe shrimp);Malacostraca (crabs, lobsters, shrimp, woodlice, etc.); Maxillopoda (barnacles, copepods, etc.); Ostracoda (seed shrimp) and Remipedia (remipedes)

7. The plant numbers **DO NOT** include species from the *1997 IUCN Red List of Threatened Plants* (Walter and Gillett 1998) as those assessments used the pre-1994 IUCN system of threat categories. Hence the numbers of threatened plants in Table 1b are much lower when compared to the 1997 results. When reporting on threatened plants, the results from the current web version of The IUCN Red List should be combined with the 1997 Plants Red List. Since there have been many taxonomic changes for plant species since 1997, careful comparison of the current and 1997 species lists will be needed when combining these results to avoid double-counting.

8. Mosses include the true mosses (Bryophyta), the hornworts (Anthoceratophyta), and liverworts (Marchantiophyta).

9. The ferns and allies include club mosses and spike mosses (Lycopodiopsida), quillworts (Isoetopsida), horsetails (Equisetopsida) and ferns (Marattiopsida, Polypodiopsida and Psilotopsida).

10. Seaweeds are included in the green algae (Chlorophyta, Charophyta), red algae (Rhodophyta), and brown algae (Ochrophyta).

11. Many of the decribed species in these groups are not elegible for assessment on the IUCN Red List as they are considered micro-organisms.

Sources for Numbers of Described Species:

Vertebrates
